# Longitudinal relationship between pre-exposure prophylaxis motivation and change in adherence among men who have sex with men in Western China

**DOI:** 10.1186/s12889-024-18729-x

**Published:** 2024-09-02

**Authors:** Bing Lin, Jiaxiu Liu, Haiying Pan, Wei He, Hong Zhang, Xiaoni Zhong

**Affiliations:** 1 School of Public Health, Chongqing Medical University, Yuzhong District, No.1 Medical College Road, Chongqing, 400016 China; 2 Research Center for Medicine and Social Development, Chongqing, China; 3 School of Medical Informatics, Chongqing Medical University, Chongqing, China

**Keywords:** Pre-exposure prophylaxis, Adherence, Motivation, Men who have sex with men, Latent profile analysis, Cohort study

## Abstract

**Introduction:**

The efficacy of pre-exposure prophylaxis (PrEP) is highly dependent on adherence, and adherence behavior is influenced by motivation. The aim of this study was to explore the longitudinal relationship between PrEP motivation and change in adherence among men who have sex with men (MSM) in Western China.

**Methods:**

From November 2019 to June 2021, we conducted a PrEP prospective cohort study. Motivation to take medicine was measured by the PrEP Motivation Scale at baseline, and was grouped into different levels of latent categories by Latent Profile Analysis (LPA). A multinomial logistic regression model was used to explore the longitudinal relationship between change in adherence (improvement, decline, no change) and different levels of PrEP motivation.

**Results:**

MSM were divided into two categories of PrEP motivation, a “high motivation group” (*n* = 506, 69.89%) and a “low motivation group” (*n* = 218, 30.11%). High PrEP motivation had no significant effect on the change in short-term adherence, however, it contributed to the improvement in long-term adherence [odds ratio (OR) = 3.028 (1.100–8.332), *p* = 0.031]. The predictive power of the adherence model was significantly enhanced with the addition of the PrEP motivation factor.

**Conclusions:**

There was a positive correlation between high PrEP motivation at baseline and an improvement in long-term adherence. Surveillance and intervention of PrEP motivation in MSM can increase their adherence, and then promote PrEP efficacy.

**Supplementary Information:**

The online version contains supplementary material available at 10.1186/s12889-024-18729-x.

## Background

Male-to-male human immunodeficiency virus (HIV) transmission is rapidly increasing and constitutes more than 25% of all newly reported HIV cases in China [1, 2]. At the same time, men who have sex with men (MSM) had the highest HIV prevalence among all key populations. Significant differences in HIV disease burden among Chinese MSM compared to the general population [2]. According to the statistics, the national HIV prevalence among MSM was 6.9% [3]. The annual incidence of HIV among MSM in China was 5 cases per 100 person-years [4]. The World Health Organization (WHO) has recommended pre-exposure prophylaxis (PrEP) as part of a comprehensive HIV prevention approach to prevent infection among MSM [5]. PrEP as a biomedical intervention has been shown to be effective in reducing HIV infection among MSM population [6, 7]. However, the success of PrEP at the population level depends heavily on individual adherence in the real-world settings. A previous study pointed out that improving MSM adherence was crucial to optimizing PrEP implementation programs [3]. Taken together, in order to effectively improve PrEP efficacy and reduce HIV transmission and prevalence, it is important to monitor and intervene early in the potential influencing factors of adherence among MSM population.

Motivation is the mental disposition or internal drive that inspires and sustains action. Previous study proposed a theoretical framework for predicting and explaining behavior, which explains how different levels of motivation regulate behavior [8]. Meanwhile, the forms of intrinsic and extrinsic motivation can also influence individual perception and behavior [9]. It is obvious that motivation is an inherent factor that causes people to develop certain behaviors or behavior change. With the emergence of more and more motivation-related studies, it is beneficial for researchers to understand and explain the relationship between motivation and behavior in greater depth, providing a theoretical basis for future behavioral guidance and management.

Currently, there have been several studies related to PrEP motivation. However, there were limited studies that focused on PrEP motivation and explored its impact on related behaviors (e.g., adherence). For example, a qualitative study assessed the motivation and interest of MSM in using PrEP in the future [10]. Another a phenomenological qualitative study showed that fear of HIV infection and the benefits of regular medical follow-up were important motivating factors for PrEP use among MSM [11]. In the study of the PrEP, the results provided insights into the relationship between motivation and PrEP intentions [12]. As we mentioned previously, motivation is an important factor influencing behavior. Most of these previous studies on PrEP motivation have been qualitative, with few studies quantitatively assessing specific relationships between PrEP motivation and adherence, and the quantitative studies lack longitudinal data and have limited ability to infer causal relationships. However, our study fills this part of the gap. The relationship between PrEP motivation and adherence in Chinese MSM needs to be further explored.

In addition, Latent Profile Analysis (LPA), as a person-centered approach, has been widely used in a number of fields, especially in psychological trait researches [13, 14]. This method allowed us to determine the heterogeneity of the study population by categorizing potential subgroups [15]. MSM in the same subgroup were homogenous, while MSM in different subgroups were heterogeneous. It could provide a better understanding of MSM population with different characteristics. LPA had the potential to develop tailored interventions for subgroups to better meet their characteristics. Meanwhile, in the variable-centered approach, it reduced the need for complex higher-order interactions between variables and resulted in concise simulations [16, 17]. In previous studies, LPA was used to analyze the potential characteristics of psychological resilience in emergency nurses, depressive symptoms in college students and their relationship with physical activity [14, 18]. The method has good applicability in psychological characterization studies. Motivation is similarly an important psychological trait variable and there is a lack of literature on the application of LPA to PrEP motivation in MSM population. Applying LPA allowed the identification of previously unobserved subgroups of population, leading to a better understanding of their hidden characteristics.

Therefore, based on data from a prospective cohort study in Western China, this study used the LPA model and aimed to investigate whether baseline PrEP motivation in the MSM population increased the likelihood of improvement in adherence and decreased the risk of decline in adherence, to further confirm the relationship between PrEP motivation and change in adherence, and to assess the predictive power of the adherence model.

## Methods

### Study design and participants

This study was a prospective cohort study conducted in Western China from November 2019 to June 2021. We recruited MSM who met the criteria in Chongqing, Sichuan, and Xinjiang in Western China with local NGOs (Non-Governmental Organizations) and peer referrals. Our study was approved by the Ethics Committee of Chongqing Medical University (Reference number: 2019001). Before participating in this research, the participants were fully informed about the purpose, significance, voluntary participation, and confidentiality of the research. Each participant signed an informed consent form. The main inclusion criteria included: (1) biological male (male at birth); (2) 18–65 years old; (3) had engaged in sex with male partners in the past 6 months; (4) HIV antigen antibody test negative; (5) willing to use medicine under guidance and follow up schedule; (6) signed informed consent.

Eligible study participants were enrolled in the PrEP cohort study. Participants took oral PrEP daily (Lamivudine and Tenofovir Disoproxil Fumarate Tablets, 300 mg/tablet), and one tablet once a day. Follow-up visits and new drug dispensations were conducted every three months.

### Measures

MSM who met the criteria were administered a basic demographic questionnaire that focuses on basic demographic characteristics, HIV-related characteristics, substance use characteristics, and the PrEP Motivation Scale.

The basic demographic characteristics included age, household registration location, ethnicity, education attainment, employment status, marital status, and monthly personal income.

There was a total of 11 variables for HIV-related characteristics of the MSM population. HIV knowledge score is measured by the HIV Knowledge Scale, which consists of 13 questions related to HIV, and a score of ≥ 11 indicates a high level of HIV knowledge [19]. Participants were asked, "Have you ever been tested and counseled for HIV before this visit?" Participants answered "yes" or "no" and their HIV testing and HIV counseling status were assessed. Participants measured their risk perception by using a score of 1 to 5 to assess their likelihood of HIV infection. 1 to 2 represented a low level of risk perception, 3 represented a moderate level, and 4 to 5 represented a high level. Previous literature mentioned that MSM may show different preferences and willingness to use PrEP and other biomedical prevention strategies based on the role of anal sex [20]. Our study included the variable "sexual role" to explore whether differences in sexual roles might lead to differences adherence to PrEP in MSM population. Sexual role was the way of performing sexual behavior with a male sexual partner, such as inserter (like "top") and receiver (like "bottom"). Participants were also asked about the number of male and female sexual partners in the last month; frequency of condom use; using condom throughout the sexual behavior; finding sexual partners though the Internet; and the history of sexually transmitted diseases (STD).

Characteristics of substance use mainly include alcohol use, recreational drug use, and engaging in commercial sex work. Substance use (alcohol use being the most common) was a common behavior among individuals taking PrEP and could influence medication adherence and engagement in HIV care and prevention [21]. Unhealthy alcohol use was a common and often unaddressed behavior associated with increased risk of acquisition and possibly decreased adherence to oral PrEP [22]. In our study, we included alcohol use because we believe that alcohol use may have an impact on PrEP adherence in MSM.

### PrEP motivation

We used the PrEP Motivation Scale (Cronbach's Alpha = 0.713) to measure PrEP motivation among MSM population at baseline [23]. The scale divided PrEP motivation into personal motivation and social motivation, using 11 items to measure (Table 1). PrEP motivation scores were based on a five-point Likert scale ("1" for "not at all" and "5" for "always"), with total scores ranging from 11 to 55 on the scale. Higher PrEP motivation scores indicate stronger motivation for prevention [24, 25].

**Table 1:** The PrEP motivation scale

	Items
Personal motivation	I think drugs make me safer, away from AIDS
	I'm scared of AIDS
	I worry that the drug has no effect^a^
	I worry about the side effects of drugs^a^
	I find it inconvenient to take the medicine^a^
	I think it’s very troublesome to take the medicine^a^
Social motivation	I'm worried that male sexual partners know I'm taking medicine^a^
	I worry that other people will discriminate me when they know I am on medicine^a^
	The doctors here are friendly to me and care for my health
	I think the doctors here discriminate against me^a^
	I trust the doctors here

^a^The item was assigned in reverse

*PrEP* pre-exposure prophylaxis, *AIDS* Acquired Immune Deficiency Syndrome

### Adherence

At each follow-up visit, adherence was measured by self-reported. When asked, “In the last two weeks, have you had any missed doses? How many days did you miss?” the participants answered “yes” or “no” and the number of days missed (0–14 days). Adherence is equal to the proportion of days of medicine-taken. All answers were checked by the researchers. In case of illogical answers, quality control and corrections were made in the study site.

**Fig. 1:**
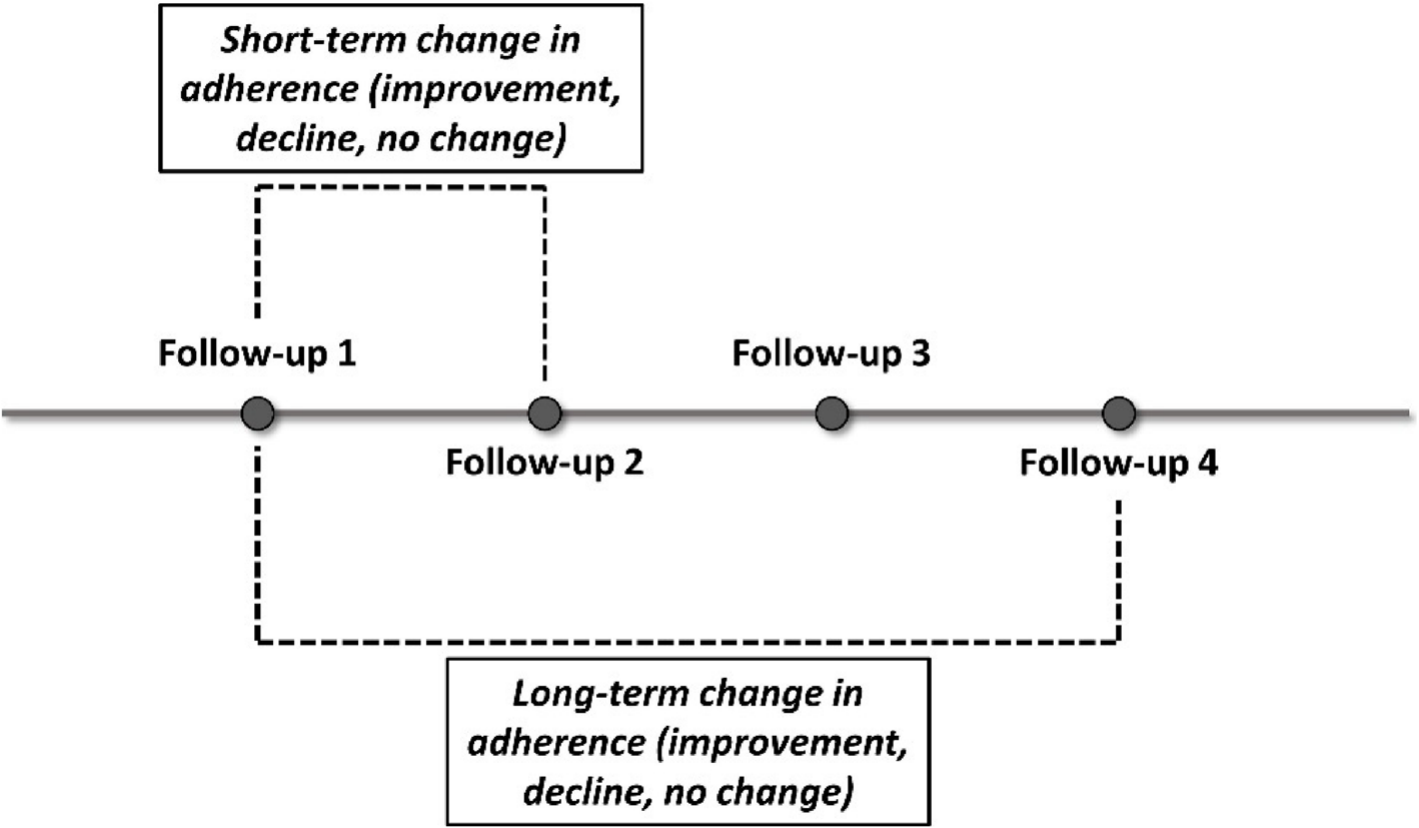
The schematic representation of long-term and short-term change in adherence

Follow-up visits were conducted every 3 months for a total of 4 visits, and we defined each follow-up visit as “Follow-up 1”, "Follow-up 2", "Follow-up 3", and "Follow-up 4". Change in short-term adherence refers to change in adherence from Follow-Up 1 to Follow-up 2 (improvement, decline, no change). Change in long-term adherence refers to change in adherence from Follow-Up 1 to Follow-up 4 (improvement, decline, no change). The schematic representation of long-term and short-term change in adherence is shown in Fig. 1.

### Statistical analysis

Latent Profile Analysis (LPA) was used to group PrEP motivation into latent categories at baseline. The model fits were estimated by AIC (Akaike), BIC (Bayesian), aBIC (Sample-Size Adjusted BIC), Entropy, VLRT (Vuong-Lo-Mendell-Rubin Likelihood Ratio Test), and BLRT (Bootstrapped Likelihood Ratio Test). The smaller values of AIC, BIC, and aBIC, Entropy ≥ 0.80, and *p*-values < 0.05 for VLRT and BLRT indicate a good model fit. The optimal latent category classification scheme was selected to compare the variability of basic demographic characteristics, HIV-related characteristics, and substance use characteristics among different latent category groups.

The relationship between different levels of PrEP motivation and changes in short-term and long-term adherence in the MSM population was explored by multinomial logistic regression models, using change in adherence (improvement, decline, no change) as the dependent variables and no change in adherence as the reference. Variables with p ≤ 0.15 were first screened by univariate analysis and included in the regression model analysis, which was estimated using odds ratio (OR) and 95% confidence interval (CI).

The predictive ability of the adherence model with the addition of PrEP motivation factor was assessed based on three indicators: AUC (area under the receiver operating characteristic curve), NRI (net reclassification index) and IDI (integrated discrimination improvement). The increase in AUC values, NRI > 0, IDI > 0, and *p*-values < 0.05 for all three indicators represent a positive enhancement of the predictive power of the model by the newly added variables. Since all independent variables had less than 10% missing values, missing values in the data were filled using the multiple fill method. The data analysis was done by Mplus software and SAS software.

## Results

**Fig. 2:**
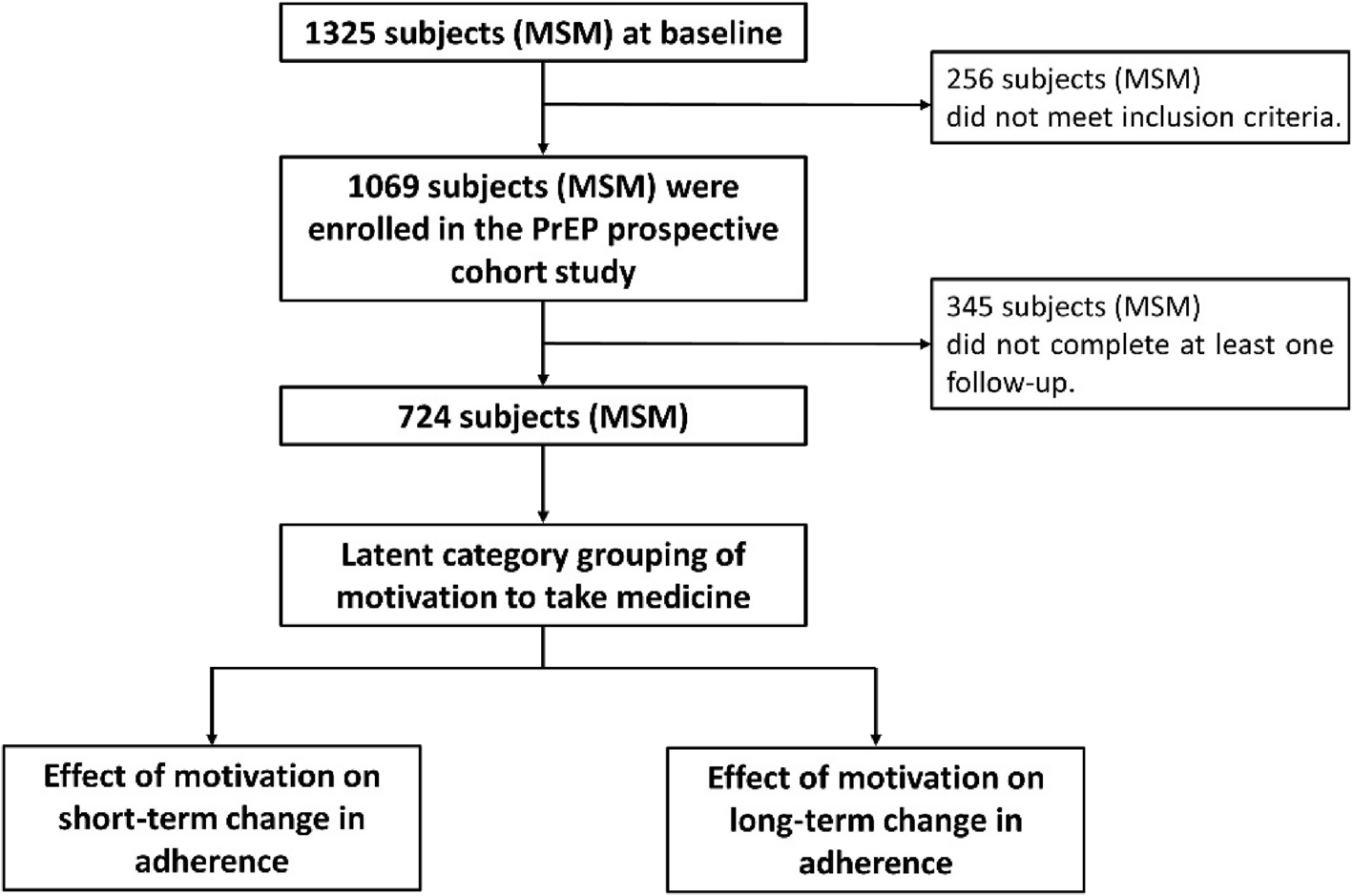
The screening flow chart of the study participants. MSM: men who have sex with men; PrEP: pre-exposure prophylaxis

We recruited a total of 1325 MSM in Western China. 256 of them did not meet the inclusion criteria and were excluded, and a total of 1069 MSM were enrolled in the PrEP prospective cohort study. A total of 724 MSM were included in the model for the Latent Profile Analysis (LPA) because 345 MSM did not complete at least one follow-up visit and data on drug adherence were missing. The screening flow chart of the study participants is shown in Fig. 2.

Then we explored the classification of latent categories from category 1 to category 5, respectively (Supplementary Table 1). According to the model fitting results, category 2 has the best model interpretability and the largest decrease in the values of indicators compared to others. Therefore, we selected the model with 2 latent categories (AIC = 25,812.95, BIC = 24,724.82, aBIC = 24,616.91, Entropy = 0.860, VLRT < 0.05, BLRT < 0.05).

218 (30.11%) MSM were assigned to latent category 1 with a mean and standard deviation of 35.43 ± 4.83 for PrEP motivation and a median and interquartile spacing of 36.00 (33.00–39.00). 506 (69.89%) MSM were assigned to latent category 2 with a mean and standard deviation of 43.66 ± 4.72 for PrEP motivation and a median and interquartile spacing of 44.00 (41.00–47.00). We defined latent category 1 as "low motivation group" and latent category 2 as "high motivation group". Comparison of the differences at baseline basic demographic characteristics, HIV-related characteristics, and substance use characteristics between the high and low motivation groups showed no statistical differences (Table 2).

**Table 2:** Differential comparison of several characteristics between the high motivation group and the low motivation group among MSM population

Variables	Total	Low motivation		High motivation		*p*-value
	*N* = 724	*N* = 218		*N* = 506		
	N	N	%	N	%	
Age						
18–25	142	43	19.73	99	19.57	0.557
25–35	329	93	42.66	236	46.64	
≥ 35	253	82	37.61	171	33.79	
Household registration location						
Urban	516	160	73.39	356	70.36	0.407
Rural	208	58	26.61	150	29.64	
Ethnicity						
Ethnic Minority	51	13	5.96	38	7.51	0.456
Ethnic Han	673	205	94.04	468	92.49	
Education attainment						
Junior high school and below	57	18	8.26	39	7.71	0.318
High school	156	43	19.72	113	22.33	
College	184	65	29.82	119	23.52	
Undergraduate training or higher	327	92	42.20	235	46.44	
Employment status						
Unemployed	54	17	7.80	37	7.31	0.934
Employed	593	179	82.11	414	81.82	
Internal student	77	22	10.09	55	10.87	
Marital status						
Single	623	182	83.49	441	87.15	0.191
Married	101	36	16.51	65	12.85	
Monthly personal income						
≤ 1000	46	11	5.05	35	6.92	0.694
1000–3000	139	46	21.10	93	18.38	
3000–5000	236	71	32.57	165	32.61	
> 5000	303	90	41.28	213	42.09	
HIV knowledge score						
< 11	393	116	53.21	277	54.74	0.704
≥ 11	331	102	46.79	229	45.26	
HIV testing						
No	49	12	5.50	37	7.31	0.374
Yes	675	206	94.50	469	92.69	
HIV counseling						
No	189	61	27.98	128	25.30	0.451
Yes	535	157	72.02	378	74.70	
Risk perception of infecting HIV						
Low level	419	118	54.13	301	59.49	0.331
Moderate level	228	77	35.32	151	29.84	
High level	77	23	10.55	54	10.67	
Sexual role						
Bottom	91	32	14.68	59	11.66	0.531
Both	372	109	50.00	263	51.98	
Top	261	77	35.32	184	36.36	
Number of male sexual partner						
0	73	20	9.17	53	10.47	0.393
1	414	133	61.01	281	55.54	
≥ 2	237	65	29.82	172	33.99	
Number of female sexual partner						
0	615	178	81.65	437	86.36	0.225
1	84	32	14.68	52	10.28	
≥ 2	25	8	3.67	17	3.36	
Frequency of condom use						
Every time	503	160	73.39	343	67.79	0.079
Sometimes or occasionally	174	41	18.81	133	26.28	
Never	47	17	7.80	30	5.93	
Using condom throughout the sexual behavior						
Yes	605	187	85.78	418	82.61	0.291
No	119	31	14.22	88	17.39	
Finding sexual partners though the Internet						
No	256	68	31.19	188	37.15	0.124
Yes	468	150	68.81	318	62.85	
History of sexually transmitted diseases (STD)						
Yes	42	13	5.96	29	5.73	0.903
No	682	205	94.04	477	94.27	
Alcohol use						
Never	315	98	44.95	217	42.89	0.506
Drinking occasionally	383	110	50.46	273	53.95	
Drinking almost every day	26	10	4.59	16	3.16	
Recreational drug use						
No	707	215	98.62	492	97.23	0.257
Yes	17	3	1.38	14	2.77	
Engaging in commercial sex work						
Yes	30	9	4.13	21	4.15	0.989
No	694	209	95.87	485	95.85	

**Fig. 3:**
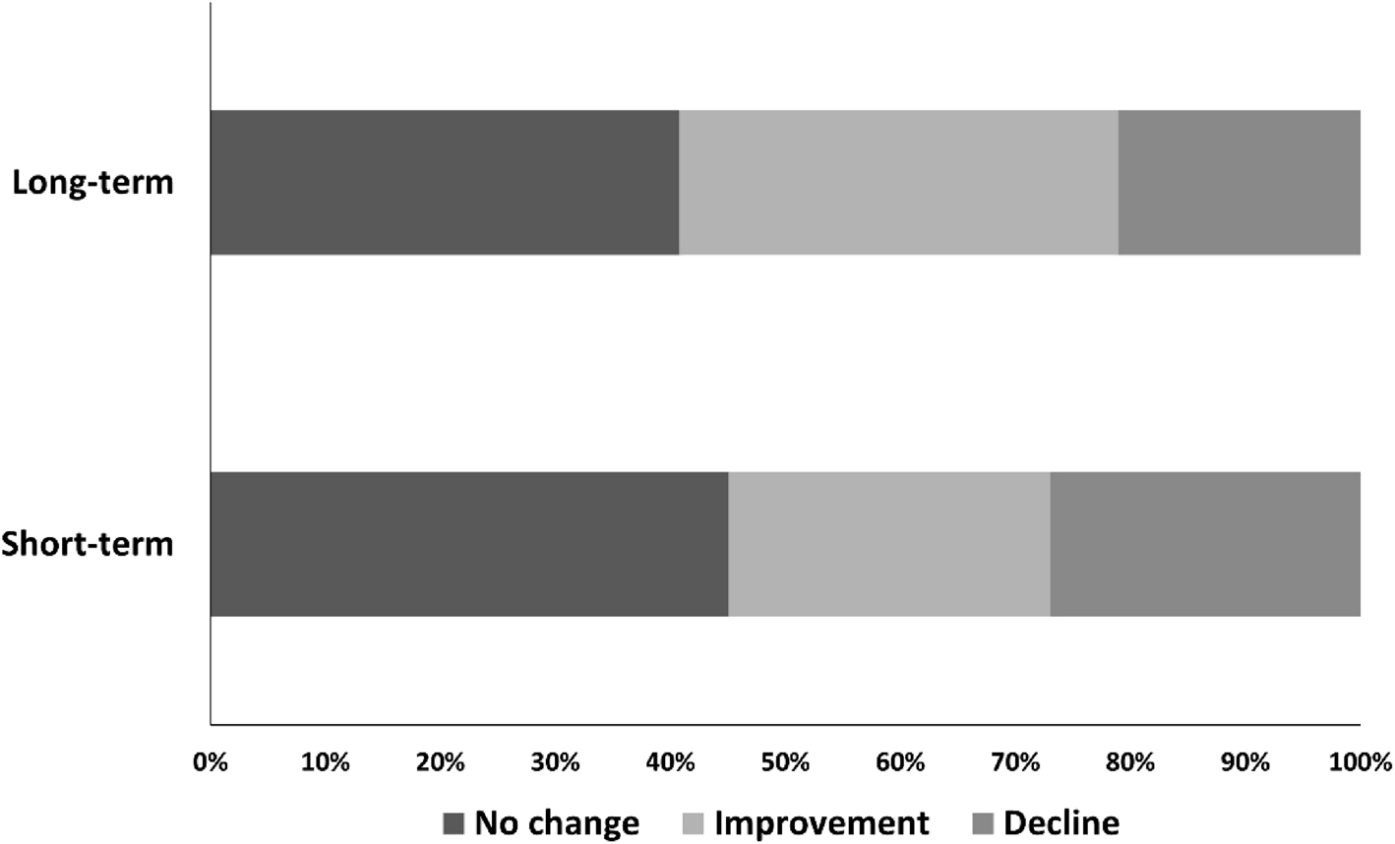
Percentage bar graphs of changes in short-term and long-term adherence

A percentage bar chart was used to describe the changes in short-term and long-term adherence (improvement, decline, no change, Fig. 3). Compared with the short-term phase, the proportion of the improvement in long-term adherence increased significantly, and the proportion of no change and decline in adherence decreased respectively (The results of the Chi-square test were shown in Supplementary Table 2). Multinomial logistic regression models were developed using changes in short-term and long-term adherence as dependent variables, respectively, and no change in adherence as reference. The process of screening variables for the multinomial logistic regression model was described in the Supplementary Table 3–6. According to the results of univariate analysis, the *p*-values of age and ethnicity were 0.096 and 0.062 for the subgroups with different long-term change in adherence, respectively. This suggested that older and Han Chinese MSM were more likely to have high adherence, so we adjusted for these in our final model. Based on the final model (Table 3), there was no significant effect of different levels of PrEP motivation on the change in short-term adherence, with *p*-values greater than 0.05. High levels of PrEP motivation contributed to the improvement in long-term adherence (OR = 3.028, 95%CI: 1.100–8.332, p = 0.031). However, high levels of PrEP motivation to reduce the risk of the decline in long-term adherence showed no significant effect (p > 0.05).

**Table 3:** Relationship between change in adherence (improvement, decline, no change) and PrEP motivation: a multinomial logistic regression model analysis

Motivation	Improvement vs. No change		Decline vs. No change	
	OR (95% CI)	*p*-value	OR (95% CI)	*p*-value
Short-term PrEP Motivation				
High	1.185 (0.699–2.009)	0.528	1.141 (0.676–1.924)	0.621
Low	Reference	-	Reference	-
Long-term PrEP Motivation				
High	3.028 (1.100–8.332)	0.031	1.976 (0.562–6.952)	0.288
Low	Reference	-	Reference	-

After univariate analysis, variables with *p* ≤ 0.15 were included in multinomial logistic regression model (Supplementary Table 3–6). In the change in short-term adherence model, variables were adjusted: HIV knowledge score (*p* = 0.070), finding sexual partners though the Internet (*p* = 0.143), engaging in commercial sex work (*p* = 0.132). In the change in long-term adherence model, variables were adjusted: age (*p* = 0.096), ethnicity (*p* = 0.062). See the supplementary file for details

*PrEP* pre-exposure prophylaxis

The conventional model refers to the model without PrEP motivation variable. In the short-term phase, the improvement and decline in adherence models showed no significant enhancement in predictive power compared to the model included PrEP motivation variable, with all *p*-values > 0.05. However, in the long-term phase, the predictive power of the improvement in adherence model increased significantly when PrEP motivation variable was added. AUC increased from 0.583 to 0.699, NRI = 0.665, IDI = 0.123, and all *p*-values < 0.05, which were statistically significant. There was no significant enhancement in predictive ability for the decline in adherence model in the long-term phase (Table 4).

**Table 4:** Comparison of the predictive power of the improvement and decline in adherence models when PrEP motivation variable was added in the short-term and long-term phase

Model	Improvement vs. No change					
	AUC		NRI		IDI	
	Estimate (95%CI)	*p*-value	Estimate (95%CI)	*p*-value	Estimate (95%CI)	*p*-value
Short term						
Conventional model	0.601 (0.537–0.665)	-	Reference	-	Reference	-
Conventional model + Motivation	0.608 (0.543–0.673)	0.537	-0.101 (-0.317–0.116)	0.405	-0.001 (-0.005–0.003)	0.641
Long term						
Conventional model	0.583 (0.465–0.700)	-	Reference	-	Reference	-
Conventional model + Motivation	0.699 (0.583–0.816)	0.036	0.665 (0.263–1.067)	0.003	0.123 (0.048–0.199)	0.001
Model	Decline vs. No change					
	AUC		NRI		IDI	
	Estimate (95%CI)	*p*-value	Estimate (95%CI)	*p*-value	Estimate (95%CI)	*p*-value
Short term						
Conventional model	0.550 (0.483–0.617)	-	Reference	-	Reference	-
Conventional model + Motivation	0.550 (0.482–0.618)	0.987	-0.061 (-0.283–0.160)	0.617	-0.001 (-0.004–0.002)	0.609
Long term						
Conventional model	0.559 (0.409–0.709)	-	Reference	-	Reference	-
Conventional model + Motivation	0.623 (0.460–0.786)	0.253	0.292 (-0.254–0.838)	0.306	0.028 (-0.014–0.070)	0.196

The conventional model refers to the model without PrEP motivation variable

*PrEP* pre-exposure prophylaxis, *AUC* area under the receiver operating characteristic curve, *NRI* net reclassification index, *IDI* integrated discrimination improvement

The results of the sensitivity analysis of other classification schemes can be found in the supplementary file (Supplementary Table 7–10). The scheme of 3C classification in LPA was selected for sensitivity analysis, and univariate and multinomial logistic regression analyses were performed. The results of the sensitivity analysis also illustrated that the classification scheme of two potential categories of PrEP motivation was the most reasonable.

## Discussion

Our findings showed that PrEP motivation was divided into two levels (high and low motivation groups) based on a PrEP prospective cohort study among MSM population in Western China, and provided evidence that high level of baseline PrEP motivation was significantly associated with the improvement in long-term adherence during follow-up.

Motivation is a key factor influencing behavior. A review published earlier showed that daily use of oral PrEP was effective, but the motivation to use drug and the perception of high risk was also of concern [26], which emphasized the importance of the motivation to take medicine. Understanding the motivation of MSM population to participate in HIV biomedical prevention strategy is also essential to increase their representativeness in studies that validate prevention strategies appropriate to their developmental characteristics and living environment [27]. PrEP can be boosted by the "motivation" of transgender and gender non-binary individuals to live an active and healthy life without fear of contracting HIV, according to a qualitative study in Southern California [28]. Four Multisite Prevention Trials, based on the their accumulated experience, also suggested fostering motivation can help support PrEP use [29]. These previous studies have highlighted the importance of motivation for PrEP, and our findings also further provided a more useful reference for PrEP motivation and adherence. According to our study, there is further evidence of the significant correlation between baseline PrEP motivation and improvement in long-term adherence among MSM population in Western China. In addition, our study also validated the predictive value of baseline PrEP motivation in terms of change in long-term adherence, improving the predictive power of adherence model.

Since motivation plays a critical role in PrEP adherence, appropriate interventions should be implemented for MSM with low levels of motivation who were screened at baseline. A brief motivational interview (MI) was found to be a feasible and acceptable method. The MI approach promoted motivation to take PrEP though a nonjudgmental discussion of the benefits and implications of taking PrEP and how this decision might align with individual’s goals. Previous research developed and evaluated a brief (15–25 min) motivational interviewing (MI)-based intervention to promote PrEP uptake. Its long-term goal was to develop a brief clinical intervention to promote acceptance of PrEP among high-risk MSM [30]. Furthermore, a pilot randomized controlled trial (RCT) also demonstrated preliminary effects of a brief behavioral motivational intervention in promoting the use of the drug among MSM who were behaviorally at risk for HIV infection [31]. Based on this, our findings were able to screen out the low-motivated MSM population and provide them with targeted motivational interventions, thereby increasing the efficiency of the interventions and promoting PrEP uptake in the high-risk population.

Prospective cohort studies of PrEP adherence would involve repeated measurements of adherence and adherence trajectories. For example, previous studies used the group-based trajectory model to construct the long-term adherence model [32], and growth mixture model (GMM) was utilized to identify individual subgroups with similar adherence trajectories [33]. However, our study used a different method than previous studies. For the longitudinal trajectory of adherence, we focused on the change in adherence and divided the changes into short-term and long-term phase to explore the impact of baseline PrEP motivation on change in adherence in the MSM population, which provides new ideas for more longitudinal analysis of adherence in the future. Notably, our results showed that PrEP motivation changed adherence in the long-term phase more significantly than in the short-term phase, suggesting that the effect of PrEP motivation on change in adherence became stronger with longer follow-up. And this is the previous longitudinal analysis of adherence could not conclude. In addition, according to our results, there were no statistically significant differences in several characteristics between high motivation group and low motivation group, suggesting that these variables did not affect the relationship between PrEP motivation and change in adherence. Moreover, PrEP motivation still had a significant impact on the change in adherence after adjusting other factors through univariate analysis. The predictive power of the long-term adherence model significantly increased when PrEP motivation variable was added. It suggests that baseline PrEP motivation can serve as a predictor of change in long-term adherence, which is very important for guiding and managing adherence. According to the PrEP Motivation Scale we use, to improve PrEP motivation in MSM population, we can consider two aspects. One the one hand, it is crucial from the perspective of the individual to improve self-efficacy in taking medicines and perceived HIV risk, as well as to comprehend the understanding of taking medicines in order to lessen worry about side effects. On the other hand, from a social perspective, we focus on the mental health of users and improve the relationship between medical professionals and patients in order to improve users' confidence in using medicines. Taken together, these findings from our study can provide additional favorable information for practice and research on the PrEP continuum of care.

We believe that there are several theories to support the effect of PrEP motivation on change in adherence: the Information-Motivation-Behavioral Skills (IMB) Model [24], the Protection Motivation Theory (PMT) [34], the Self-Determination Theory (SDT) [8] and Theory of Planned Behavior [35]. However, these theories are all related to motivation but posit slightly different and nuanced ways of understanding motivation and its impact on health behavior change. For example, SDT was typically used and thought of in terms of individual behavior change, often alongside motivational interviewing [36]. We believe that IMB can most accurately describe the theory of how motivation and PrEP adherence may operate in the MSM population. This model, first proposed by Fisher, is a theoretical model of healthy behavior. Information related to prevention, motivation related to prevention, and behavioral skills required for prevention were the important components of the model and were highly generalizable determinants of HIV prevention behavior [37, 38]. IMB model has been shown to be effective in enhancing HIV care, suggesting that motivation can increase the willingness of social workers to change clinical practice [39]. The IMB model also has been proven useful in promoting the willingness to use PrEP [40]. Previous study among Black MSM and transgender women in New York City found that motivational and behavioral skills screening can be used to identify where additional support is needed to improve PrEP adherence [41]. Based on the IMB model, the relationship between PrEP motivation and adherence behavior in MSM population in our study was explained, facilitating a better understanding of the relationship between motivation and behavior, and thus clarifying the direction of guidance, intervention, and management. In summary, the relevant theories on motivation and behavior not only provide a solid basis for our study, but also explain our findings well.

In our study we acknowledged that there were still some limitations. First, we excluded the MSM population that did not complete at least one adherence follow-up visit. The results of our analysis showed that the means and standard deviations of the PrEP motivation scores were 40.32 ± 5.95 and 41.19 ± 6.07 for the excluded and included groups, respectively; the median and interquartile range were 41.00 (36.00–45.00) and 42.00 (37.00–46.00), respectively. The difference in baseline PrEP motivation scores between the two groups was not significant, and we believe that it has a small potential impact on the results (Supplementary Table 11). Meanwhile, we are performing Latent profile analysis (LPA) based on the included data, which also avoids the effect of the excluded data on the classification of latent categories. Secondly, although the PrEP Motivation Scale (Cronbach's alpha = 0.713) has been validated in previous research [23], the scale is currently only used in the MSM population, and whether it is applicable to a broader population or includes additional motivational factors needs to be further explored in future research. In addition, for MSM with low levels of motivation, our study did not conduct a motivational interviewing-based intervention to explain the relationship between motivation and adherence behaviors more deeply. In future research and practice, motivational interviewing-based interventions should be included as a way to illustrate the effects of motivation on behavior. Lastly, adherence in this study was measured by self-reported, which may be overestimated due to recall bias and social desirability bias. Meanwhile, only employing 1 item to assess adherence was one of the limitations of our study. If conditions permit, we should also consider employing objective adherence measurements. However, previous studies have also pointed out that self-reported data may be reliable [42, 43].

## Conclusion

In conclusion, our study suggests a positive association between high levels of baseline PrEP motivation and improvement in long-term adherence in the MSM population in Western China. Considering that PrEP efficacy is highly dependent on adherence and motivation is a modifiable subjective psychological characteristic, early monitoring, and intervention of PrEP motivation in MSM population may be important to improve adherence and enhance PrEP efficacy.

### Supplementary Information

Supplementary Material.

## Data Availability

For the privacy of MSM population, the dataset supporting the conclusion of this article is available upon reasonable request from the corresponding author.
