# Supplementary material for: Longitudinal relationship between pre-exposure prophylaxis motivation and change in adherence among men who have sex with men in Western China
Source: BMC Public Health. 2024 Sep 2;24:1271. doi: 10.1186/s12889-024-18729-x (PMC11367776; doi:10.1186/s12889-024-18729-x)
Supplement: Supplementary file 1 — Supplementary Material. [file 12889_2024_18729_MOESM1_ESM.docx]

**Longitudinal relationship between pre-exposure prophylaxis motivation and change in adherence among men who have sex with men in Western China**

**Supplementary file**

**1. Latent Profile Analysis (LPA) results for baseline PrEP motivation in MSM population**

According to the results in **Supplementary Table 1**, the indicators of 2C, 3C, and 4C all met the criteria for classification. The final classification results always serve the analysis of the paper. In the original paper, we considered 2C as the most appropriate classification scheme. For the 4C classification scheme, although the indicators are qualified, the percentage of one of the categories is too small (10.21%) to facilitate the analysis. Therefore, we believe that the classification scheme of 4C is not as good as the first two schemes. For the classification scheme of 3C, we will conduct a sensitivity analysis subsequently.

Supplementary Table 1. Summary of model fit information by potential profile analysis for each potential category

|  | AIC | BIC | aBIC | Entropy | VLRT | BLRT | Class probability |
| --- | --- | --- | --- | --- | --- | --- | --- |
| 1C | 25812.95 | 25913.82 | 25843.96 | - | - | - | 1 |
| 2C | 24568.99 | 24724.87 | 24616.91 | 0.860 | <.001 | <.001 | 0.3011/0.6989 |
| 3C | 23748.80 | 23959.70 | 23813.64 | 0.908 | <.001 | <.001 | 0.2403/0.2113/0.5484 |
| 4C | 23050.38 | 23316.29 | 23132.13 | 0.928 | 0.007 | <.001 | 0.1658/0.2376/0.4945/0.1021 |
| 5C | 22465.20 | 22786.14 | 22563.87 | 0.938 | 0.064 | <.001 | 0.0497/0.1077/0.4876/0.2307/0.1243 |

AIC: Akaike; BIC: Bayesian; aBIC: Sample-Size Adjusted BIC; VLRT: Vuong-Lo-Mendell-Rubin Likelihood Ratio Test; BLRT: Bootstrapped Likelihood Ratio Test.

**2. Chi-square test results of short-term and long-term change in adherence (improvement, decline, no change)**

A percentage bar chart was used to describe the changes in short-term and long-term adherence (improvement, decline, no change, Figure 3). Compared with the short-term phase, the proportion of the improvement in long-term adherence increased significantly, and the proportion of no change and decline in adherence decreased respectively (The results of the Chi-square test were shown in **Supplementary Table 2**). No change was reduced from 45.09% to 35.71% and Decline was reduced from 26.95% to 19.39%. However, the percentage of adherence Improvement increased from 27.96% to 44.90%. Meanwhile, the results of the Chi-square test were statistically different with a p-value of 0.0051.

Supplementary Table 2. Chi-square test results of short-term and long-term change in adherence (improvement, decline, no change)

| Change in adherence | Short-term | | Long-term | | p-value |
| --- | --- | --- | --- | --- | --- |
|  | N | % | N | % |  |
| No change | 179 | 45.09 | 35 | 35.71 | 0.0051 |
| Improvement | 111 | 27.96 | 44 | 44.90 |  |
| Decline | 107 | 26.95 | 19 | 19.39 |  |

**3. Univariate analysis of short-term change in adherence (improvement, decline, no change) for each variable**

We compared the variability of the variables across groups for short-term change in adherence. Variables with p-values≤0.15 were included in the multinomial logistic regression model. According to the results in **Supplementary Table 3**, p-values≤0.15 for the variables HIV knowledge score (p=0.070), finding sexual partners though the Internet (p=0.143), and engaging in commercial sex work (p=0.132) needed to be included in the multinomial logistic regression. PrEP motivation factor was not statistically different in the different groups of adherence (p=0.649). However, to explore the effect of PrEP motivation on short-term change in adherence, this variable was still included in the multinomial logistic regression.

Supplementary Table 3. Univariate analysis of short-term change in adherence (improvement, decline, no change) for each variable

| Variables | Change in Adherence | | | | | | p-value |
| --- | --- | --- | --- | --- | --- | --- | --- |
|  | No change | | Improvement | | Decline | |  |
|  | N | % | N | % | N | % |  |
| Age | | | | | | | |
| 18-25 | 23 | 12.84 | 20 | 18.02 | 21 | 19.63 | 0.196 |
| 25-35 | 93 | 51.96 | 48 | 43.24 | 41 | 38.32 |  |
| ≥35 | 63 | 35.20 | 43 | 38.74 | 45 | 42.05 |  |
| Household registration location | | | | | | | |
| Urban | 133 | 74.30 | 85 | 76.58 | 85 | 79.44 | 0.612 |
| Rural | 46 | 25.70 | 26 | 23.42 | 22 | 20.56 |  |
| Ethnicity | | | | | | | |
| Ethnic Minority | 10 | 5.59 | 8 | 7.21 | 12 | 11.21 | 0.216 |
| Ethnic Han | 169 | 94.41 | 103 | 92.79 | 95 | 88.79 |  |
| Education attainment | | | | | | | |
| Junior high school and below | 9 | 5.03 | 8 | 7.21 | 7 | 6.54 | 0.541 |
| High school | 30 | 16.76 | 27 | 24.32 | 19 | 17.76 |  |
| College | 50 | 27.93 | 33 | 29.73 | 29 | 27.10 |  |
| Undergraduate training or higher | 90 | 50.28 | 43 | 38.74 | 52 | 48.60 |  |
| Employment status | | | | | | | |
| Unemployed | 9 | 5.03 | 12 | 10.81 | 6 | 5.61 | 0.235 |
| Employed | 153 | 85.47 | 92 | 82.88 | 95 | 88.78 |  |
| Internal student | 17 | 9.50 | 7 | 6.31 | 6 | 5.61 |  |
| Marital status | | | | | | | |
| Single | 158 | 88.27 | 93 | 83.78 | 91 | 85.05 | 0.521 |
| Married | 21 | 11.73 | 18 | 16.22 | 16 | 14.95 |  |
| Monthly personal income | | | | | | | |
| ≤1000 | 10 | 5.59 | 8 | 7.21 | 4 | 3.74 | 0.849 |
| 1000-3000 | 32 | 17.88 | 22 | 19.82 | 24 | 22.43 |  |
| 3000-5000 | 62 | 34.63 | 37 | 33.33 | 40 | 37.38 |  |
| >5000 | 75 | 41.90 | 44 | 39.64 | 39 | 36.45 |  |
| HIV knowledge score | | | | | | | |
| <11 | 114 | 63.69 | 56 | 50.45 | 59 | 55.14 | 0.070 |
| ≥11 | 65 | 36.31 | 55 | 49.55 | 48 | 44.86 |  |
| HIV testing | | | | | | | |
| No | 9 | 5.03 | 6 | 5.41 | 6 | 5.61 | 0.976 |
| Yes | 170 | 94.97 | 105 | 94.59 | 101 | 94.39 |  |
| HIV counseling | | | | | | | |
| No | 47 | 26.26 | 28 | 25.23 | 27 | 25.23 | 0.973 |
| Yes | 132 | 73.74 | 83 | 74.77 | 80 | 74.77 |  |
| Risk perception of infecting HIV | | | | | | | |
| Low level | 100 | 55.87 | 72 | 64.86 | 59 | 55.14 | 0.521 |
| Moderate level | 61 | 34.08 | 28 | 25.23 | 36 | 33.64 |  |
| High level | 18 | 10.05 | 11 | 9.91 | 12 | 11.22 |  |
| Sexual role | | | | | | | |
| Bottom | 21 | 11.73 | 16 | 14.41 | 17 | 15.89 | 0.363 |
| Both | 94 | 52.52 | 64 | 57.66 | 49 | 45.79 |  |
| Top | 64 | 35.75 | 31 | 27.93 | 41 | 38.32 |  |
| Number of male sexual partner | | | | | | | |
| 0 | 14 | 7.82 | 9 | 8.11 | 9 | 8.41 | 0.987 |
| 1 | 98 | 54.75 | 64 | 57.66 | 59 | 55.14 |  |
| ≥2 | 67 | 37.43 | 38 | 34.23 | 39 | 36.45 |  |
| Number of female sexual partner | | | | | | | |
| 0 | 159 | 88.83 | 91 | 81.98 | 92 | 85.98 | 0.483 |
| 1 | 14 | 7.82 | 16 | 14.41 | 12 | 11.21 |  |
| ≥2 | 6 | 3.35 | 4 | 3.61 | 3 | 2.80 |  |
| Frequency of condom use | | | | | | | |
| Every time | 126 | 70.39 | 67 | 60.36 | 79 | 73.83 | 0.195 |
| Sometimes or occasionally | 41 | 22.91 | 31 | 27.93 | 22 | 20.56 |  |
| Never | 12 | 6.70 | 13 | 11.71 | 6 | 5.61 |  |
| Using condom throughout the sexual behavior | | | | | | | |
| Yes | 142 | 79.33 | 88 | 79.28 | 90 | 84.11 | 0.562 |
| No | 37 | 20.67 | 23 | 20.72 | 17 | 15.89 |  |
| Finding sexual partners though the Internet | | | | | | | |
| No | 57 | 31.84 | 48 | 43.24 | 38 | 35.51 | 0.143 |
| Yes | 122 | 68.16 | 63 | 56.76 | 69 | 64.49 |  |
| History of sexually transmitted diseases (STD) | | | | | | | |
| Yes | 13 | 7.26 | 9 | 8.11 | 6 | 5.61 | 0.763 |
| No | 166 | 92.74 | 102 | 91.89 | 101 | 94.39 |  |
| Alcohol use | | | | | | | |
| Never | 79 | 44.14 | 55 | 49.55 | 39 | 36.45 | 0.364 |
| Drinking occasionally | 94 | 52.51 | 54 | 48.65 | 65 | 60.75 |  |
| Drinking almost every day | 6 | 3.35 | 2 | 1.80 | 3 | 2.80 |  |
| Recreational drug use | | | | | | | |
| No | 175 | 97.77 | 107 | 96.40 | 106 | 99.07 | 0.433 |
| Yes | 4 | 2.23 | 4 | 3.36 | 1 | 0.93 |  |
| Engaging in commercial sex work | | | | | | | |
| Yes | 6 | 3.35 | 1 | 0.90 | 6 | 5.61 | 0.132 |
| No | 173 | 96.65 | 110 | 99.10 | 101 | 94.69 |  |
| PrEP motivation | | | | | | | |
| Low | 59 | 32.96 | 31 | 27.93 | 32 | 29.91 | 0.649 |
| High | 120 | 67.04 | 80 | 72.07 | 75 | 70.09 |  |

MSM: men who have sex with men; HIV: human immunodeficiency virus; STD: sexually transmitted disease; PrEP: pre-exposure prophylaxis.

The results of the multinomial logistic regression are shown in **Supplementary Table 4**. According to the results, it can be concluded that HIV knowledge score ≥11 promotes an increase in short-term adherence (OR=1.739, 95% CI=1.070-2.826, p=0.025). All remaining variables had no effect on the change in short-term adherence.

Supplementary Table 4. Results of multinomial logistic regression of short-term change in adherence (improvement, decline, no change)

| Variables | Improvement vs. No change | | Decline vs. No change | |
| --- | --- | --- | --- | --- |
|  | OR (95% CI) | p-value | OR (95% CI) | p-value |
| HIV knowledge score | | | | |
| <11 | Reference | - | Reference | - |
| ≥11 | 1.739 (1.070-2.826) | 0.025 | 1.395 (0.854-2.278) | 0.184 |
| Finding sexual partners though the Internet | | |  | |
| No | Reference | - | Reference | - |
| Yes | 0.635 (0.387-1.044) | 0.073 | 0.864 (0.519-1.438) | 0.573 |
| Engaging in commercial sex work | | | | |
| Yes | Reference | - | Reference | - |
| No | 4.167 (0.488-35.573) | 0.192 | 0.617 (0.192-1.984) | 0.418 |
| PrEP motivation | | | | |
| Low | Reference | - | Reference | - |
| High | 1.185 (0.699-2.009) | 0.528 | 1.141 (0.676-1.924) | 0.621 |

HIV: human immunodeficiency virus; PrEP: pre-exposure prophylaxis.

**4. Univariate analysis of long-term change in adherence (improvement, decline, no change) for each variable**

The variability of each variable was compared across groups of long-term change in adherence, and variables with p-values≤0.15 were included in a multinomial logistic regression model. According to the results in **Supplementary Table 5**, three variables, age (p=0.096), ethnicity (p=0.062), and PrEP motivation (p=0.079), with p-values ≤0.15 needed to be included in the multinomial logistic regression.

Supplementary Table 5. Univariate analysis of long-term change in adherence (improvement, decline, no change) for each variable

| Variables | Change in Adherence | | | | | | p-value |
| --- | --- | --- | --- | --- | --- | --- | --- |
|  | No change | | Improvement | | Decline | |  |
|  | N | % | N | % | N | % |  |
| Age | | | | | | | |
| 18-25 | 2 | 5.72 | 4 | 9.09 | 1 | 5.26 | 0.096 |
| 25-35 | 20 | 57.14 | 14 | 31.82 | 12 | 63.16 |  |
| ≥35 | 13 | 37.14 | 26 | 59.09 | 6 | 31.58 |  |
| Household registration location | | | | | | | |
| Urban | 30 | 85.71 | 35 | 79.55 | 15 | 78.95 | 0.737 |
| Rural | 5 | 14.29 | 9 | 20.45 | 4 | 21.05 |  |
| Ethnicity | | | | | | | |
| Ethnic Minority | 1 | 2.86 | 1 | 2.27 | 3 | 15.79 | 0.062 |
| Ethnic Han | 34 | 97.14 | 43 | 97.73 | 16 | 84.21 |  |
| Education attainment | | | | | | | |
| Junior high school and below | 4 | 11.43 | 3 | 6.81 | 0 | 0.00 | 0.607 |
| High school | 6 | 17.14 | 14 | 31.82 | 5 | 26.32 |  |
| College | 8 | 22.86 | 10 | 22.73 | 6 | 31.58 |  |
| Undergraduate training or higher | 17 | 48.57 | 17 | 38.64 | 8 | 42.10 |  |
| Employment status | | | | | | | |
| Unemployed | 5 | 14.29 | 7 | 15.91 | 0 | 0.00 | 0.240 |
| Employed | 27 | 77.14 | 34 | 77.27 | 19 | 100.00 |  |
| Internal student | 3 | 8.57 | 3 | 6.82 | 0 | 0.00 |  |
| Marital status | | | | | | | |
| Single | 33 | 94.29 | 40 | 90.91 | 17 | 89.47 | 0.798 |
| Married | 2 | 5.71 | 4 | 9.09 | 2 | 10.53 |  |
| Monthly personal income | | | | | | | |
| ≤1000 | 2 | 5.71 | 2 | 4.55 | 0 | 0.00 | 0.823 |
| 1000-3000 | 6 | 17.14 | 7 | 15.91 | 6 | 31.58 |  |
| 3000-5000 | 12 | 34.29 | 18 | 40.91 | 6 | 31.58 |  |
| >5000 | 15 | 42.86 | 17 | 38.63 | 7 | 36.84 |  |
| HIV knowledge score | | | | | | | |
| <11 | 19 | 54.29 | 21 | 47.73 | 7 | 36.84 | 0.472 |
| ≥11 | 16 | 45.71 | 23 | 52.27 | 12 | 63.16 |  |
| HIV testing | | | | | | | |
| No | 4 | 11.43 | 2 | 4.55 | 0 | 0.00 | 0.334 |
| Yes | 31 | 88.57 | 42 | 95.45 | 19 | 100.00 |  |
| HIV counseling | | | | | | | |
| No | 7 | 20.00 | 14 | 31.82 | 4 | 21.05 | 0.431 |
| Yes | 28 | 80.00 | 30 | 68.18 | 15 | 78.95 |  |
| Risk perception of infecting HIV | | | | | | | |
| Low level | 16 | 45.71 | 25 | 56.82 | 12 | 63.16 | 0.682 |
| Moderate level | 14 | 40.00 | 16 | 36.36 | 6 | 31.58 |  |
| High level | 5 | 14.29 | 3 | 6.82 | 1 | 5.26 |  |
| Sexual role | | | | | | | |
| Bottom | 3 | 8.57 | 4 | 9.09 | 1 | 5.26 | 0.971 |
| Both | 22 | 62.86 | 25 | 56.82 | 11 | 57.89 |  |
| Top | 10 | 28.57 | 15 | 34.09 | 7 | 36.85 |  |
| Number of male sexual partner | | | | | | | |
| 0 | 5 | 14.29 | 8 | 18.18 | 0 | 0.00 | 0.350 |
| 1 | 20 | 57.14 | 26 | 59.09 | 13 | 68.42 |  |
| ≥2 | 10 | 28.57 | 10 | 22.73 | 6 | 31.58 |  |
| Number of female sexual partner | | | | | | | |
| 0 | 28 | 80.00 | 38 | 86.36 | 18 | 94.74 | 0.715 |
| 1 | 4 | 11.43 | 3 | 6.82 | 0 | 0.00 |  |
| ≥2 | 3 | 8.57 | 3 | 6.82 | 1 | 5.26 |  |
| Frequency of condom use | | | | | | | |
| Every time | 24 | 68.57 | 31 | 70.45 | 15 | 78.95 | 0.886 |
| Sometimes or occasionally | 8 | 22.86 | 10 | 22.73 | 4 | 21.05 |  |
| Never | 3 | 8.57 | 3 | 6.82 | 0 | 0.00 |  |
| Using condom throughout the sexual behavior | | | | | | | |
| Yes | 24 | 68.57 | 33 | 75.00 | 16 | 84.21 | 0.450 |
| No | 11 | 31.43 | 11 | 25.00 | 3 | 15.79 |  |
| Finding sexual partners though the Internet | | | | | | | |
| No | 12 | 34.29 | 18 | 40.91 | 7 | 36.84 | 0.830 |
| Yes | 23 | 65.71 | 26 | 59.09 | 12 | 63.16 |  |
| History of sexually transmitted diseases (STD) | | | | | | | |
| Yes | 2 | 5.71 | 2 | 4.55 | 3 | 15.79 | 0.256 |
| No | 33 | 94.29 | 42 | 95.45 | 16 | 84.21 |  |
| Alcohol use | | | | | | | |
| Never | 19 | 54.29 | 24 | 54.55 | 8 | 42.11 | 0.827 |
| Drinking occasionally | 15 | 42.86 | 19 | 43.18 | 10 | 52.63 |  |
| Drinking almost every day | 1 | 2.85 | 1 | 2.27 | 1 | 5.26 |  |
| Recreational drug use | | | | | | | |
| No | 35 | 100.00 | 43 | 97.73 | 19 | 100.00 | 0.538 |
| Yes | 0 | 0.00 | 1 | 2.27 | 0 | 0.00 |  |
| Engaging in commercial sex work | | | | | | | |
| Yes | 2 | 5.71 | 0 | 0.00 | 0 | 0.00 | 0.161 |
| No | 33 | 94.29 | 44 | 100.00 | 19 | 100.00 |  |
| PrEP motivation | | | | | | | |
| Low | 16 | 45.71 | 10 | 22.73 | 5 | 26.32 | 0.079 |
| High | 19 | 54.29 | 34 | 77.27 | 14 | 73.68 |  |

MSM: men who have sex with men; HIV: human immunodeficiency virus; STD: sexually transmitted disease; PrEP: pre-exposure prophylaxis.

The results of the multinomial logistic regression are shown in **Supplementary Table 6**. According to the result, high levels of PrEP motivation promoted an increase in short-term adherence (OR=3.028, 95% CI=1.100-8.332, p=0.031). All remaining variables had no effect on the change in short-term adherence.

Supplementary Table 6. Results of multinomial logistic regression of long-term change in adherence (improvement, decline, no change)

| Variables | Improvement vs. No change | | Decline vs. No change | |
| --- | --- | --- | --- | --- |
|  | OR (95% CI) | p-value | OR (95% CI) | p-value |
| Age | | | | |
| 18-25 | Reference | - | Reference | - |
| 25-35 | 0.401 (0.061-2.631) | 0.091 | 1.798 (0.124-26.051) | 0.642 |
| ≥35 | 1.197 (0.181-7.916) | 0.288 | 1.546 (0.097-24.580) | 0.867 |
| Ethnicity | | | | |
| Ethnic Minority | Reference | - | Reference | - |
| Ethnic Han | 1.719 (0.095-31.155) | 0.713 | 0.194 (0.017-2.190) | 0.184 |
| PrEP motivation | | | | |
| Low | Reference | - | Reference | - |
| High | 3.028 (1.100-8.332) | 0.031 | 1.976 (0.562-6.952) | 0.288 |

PrEP: pre-exposure prophylaxis.

**4. Sensitivity analysis**

The 3C classification scheme was also reasonable based on the results of the LPA. Therefore, we subjected the three potential categories of PrEP motivation to sensitivity analysis. The LPA divided PrEP motivation into three potential categories with motivation scores of 37.72±4.23, 35.23±4.75, and 45.13±3.83, respectively; we named these three groups as the moderate PrEP motivation group (N=174), the low motivation group (N=153), and the high motivation group (N=397), respectively. Since none of the other variables changed, we only present the results for PrEP motivation in the univariate analysis (Univariate analysis of other variables of short-term change in adherence and long-term change in adherence were showing in **Supplementary Table 3 and Supplementary Table 5**). The univariate analysis for PrEP motivation is shown in **Supplementary Table 7**. Based on the results, the PrEP motivation variable was not statistically different across subgroups of adherence (p=0.349). Taking into multinomial logistic regression (**Supplementary Table 8**), HIV knowledge score ≥11 was able to promote an increase in short-term adherence (OR=1.744, 95% CI=1.073-2.236, p=0.025). None of the remaining variables influenced the short-term change in adherence. This result agreed with the results of the classification of the two potential categories in the main text.

At the same time, the variability of PrEP motivation was compared across groups for long-term change in adherence (**Supplementary Table 9**). We obtained that PrEP motivation variable was significantly different (p=0.047). Taking the new grouping of PrEP motivation variable into a multinomial logistic regression model of long-term change in adherence, we obtained that all variables were not able to influence adherence (**Supplementary Table 10**). We believe that the difference between the scores of the moderate motivation group (37.72±4.23) and the low motivation group (35.23±4.75) may be too small to show the effect of motivation on adherence behavior. After sensitivity analysis, we concluded that the classification scheme of two potential categories of PrEP motivation was the most reasonable.

Supplementary Table 7. Univariate analysis of PrEP motivation in each group of short-term change in adherence

| Variables | Change in Adherence | | | | | | p-value |
| --- | --- | --- | --- | --- | --- | --- | --- |
|  | No change | | Improvement | | Decline | |  |
|  | N | % | N | % | N | % |  |
| PrEP motivation | | | | | | | |
| Moderate | 48 | 26.82 | 25 | 22.52 | 23 | 21.50 | 0.349 |
| Low | 36 | 20.11 | 24 | 21.62 | 32 | 29.90 |  |
| High | 95 | 53.07 | 62 | 55.86 | 52 | 48.60 |  |

PrEP: pre-exposure prophylaxis.

Supplementary Table 8. Results of multinomial logistic regression of short-term change in adherence (improvement, decline, no change): Sensitivity analysis

| Variables | Improvement vs. No change | | Decline vs. No change | |
| --- | --- | --- | --- | --- |
|  | OR (95% CI) | p-value | OR (95% CI) | p-value |
| HIV knowledge score | | | | |
| <11 | Reference | - | Reference | - |
| ≥11 | 1.744 (1.073-2.236) | 0.025 | 1.365 (0.833-2.236) | 0.216 |
| Finding sexual partners though the Internet | | |  | |
| No | Reference | - | Reference | - |
| Yes | 0.630 (0.383-1.037) | 0.069 | 0.897 (0.537-1.498) | 0.678 |
| Engaging in commercial sex work | | | | |
| Yes | Reference | - | Reference | - |
| No | 4.267 (0.500-36.444) | 0.185 | 0.641 (0.198-2.074) | 0.458 |
| PrEP motivation | | | | |
| Moderate | 0.876 (0.425-1.806) | 0.595 | 0.563 (0.280-1.129) | 0.237 |
| Low | Reference | - | Reference | - |
| High | 1.053 (0.567-1.955) | 0.633 | 0.639 (0.355-1.151) | 0.516 |

HIV: human immunodeficiency virus; PrEP: pre-exposure prophylaxis.

Supplementary Table 9. Univariate analysis of PrEP motivation in each group of long-term change in adherence

| Variables | Change in Adherence | | | | | | p-value |
| --- | --- | --- | --- | --- | --- | --- | --- |
|  | No change | | Improvement | | Decline | |  |
|  | N | % | N | % | N | % |  |
| PrEP motivation | | | | | | | |
| Moderate | 14 | 40.00 | 12 | 27.27 | 5 | 26.32 | 0.047 |
| Low | 1 | 2.86 | 2 | 4.55 | 5 | 26.32 |  |
| High | 20 | 57.14 | 30 | 68.18 | 9 | 47.37 |  |

PrEP: pre-exposure prophylaxis.

Supplementary Table 10. Results of multinomial logistic regression of long-term change in adherence (improvement, decline, no change): Sensitivity analysis

| Variables | Improvement vs. No change | | Decline vs. No change | |
| --- | --- | --- | --- | --- |
|  | OR (95% CI) | p-value | OR (95% CI) | p-value |
| Age | | | | |
| 18-25 | Reference | - | Reference | - |
| 25-35 | 0.333 (0.052-2.147) | 0.065 | 1.184 (0.081-17.255) | 0.854 |
| ≥35 | 0.977 (0.152-6.269) | 0.370 | 1.041 (0.066-16.547) | 0.959 |
| Ethnicity | | | | |
| Ethnic Minority | Reference | - | Reference | - |
| Ethnic Han | 1.593 (0.087-29.193) | 0.753 | 0.168 (0.013-2.117) | 0.168 |
| PrEP motivation | | | | |
| Moderate | 0.382 (0.029-4.996) | 0.297 | 0.086 (0.008-0.954) | 0.121 |
| Low | Reference | - | Reference | - |
| High | 0.712 (0.058-8.768) | 0.845 | 0.087 (0.008-0.898) | 0.106 |

PrEP: pre-exposure prophylaxis.

**5. Comparison of the inclusion and exclusion groups**

Comparing basic information between the inclusion and exclusion groups (**Supplementary Table 11**), we obtained that age (p<0.001), household registration location (p=0.002), ethnicity (p=0.028), education attainment (p=0.007), and monthly personal income (p=0.039), these variables were statistically different between the two groups. Since our study required the inclusion of adherence data with at least one follow-up visit, we excluded MSM who were younger, rural, minority, less educated, and with lower income levels, which could potentially affect the study results. MSM population is particular, and the cohort management of this population is certainly subject to the problem of lost-follow up, which we cannot avoid. We could only strengthen reminders and management during the study to reduce missed visits of the study subjects. Meanwhile, for the PrEP motivation variable, there was no statistical difference between the inclusion and exclusion groups (p=0.057).

Supplementary Table 11. Comparison of basic information between the inclusion and exclusion groups

|  | Total | Exclusion group | | Inclusion group | | p-value |
| --- | --- | --- | --- | --- | --- | --- |
|  | N | N | % | N | % |  |
| Age | | | | | | |
| 18-25 | 247 | 105 | 30.52 | 142 | 19.62 | <0.001 |
| 25-35 | 476 | 147 | 42.74 | 329 | 45.44 |  |
| ≥35 | 345 | 92 | 26.74 | 253 | 34.94 |  |
| Household registration location | | | | | | |
| Urban | 728 | 212 | 61.81 | 516 | 71.27 | 0.002 |
| Rural | 339 | 131 | 38.19 | 208 | 28.73 |  |
| Ethnicity | | | | | | |
| Ethnic Minority | 89 | 38 | 11.01 | 51 | 7.04 | 0.028 |
| Ethnic Han | 980 | 307 | 88.99 | 673 | 92.96 |  |
| Education attainment | | | | | | |
| Junior high school and below | 107 | 50 | 14.54 | 57 | 7.87 | 0.007 |
| High school | 232 | 76 | 22.09 | 156 | 21.55 |  |
| College | 261 | 77 | 22.38 | 184 | 25.41 |  |
| Undergraduate training or higher | 468 | 141 | 40.99 | 327 | 45.17 |  |
| Employment status | | | | | | |
| Unemployed | 91 | 37 | 10.85 | 54 | 7.46 | 0.065 |
| Employed | 122 | 45 | 13.20 | 77 | 10.64 |  |
| Internal student | 852 | 259 | 75.95 | 593 | 81.90 |  |
| Marital status | | | | | | |
| Single | 913 | 290 | 84.55 | 623 | 86.05 | 0.515 |
| Married | 154 | 53 | 15.45 | 101 | 13.95 |  |
| Monthly personal income | | | | | | |
| ≤1000 | 84 | 38 | 11.11 | 46 | 6.35 | 0.039 |
| 1000-3000 | 209 | 70 | 20.47 | 139 | 19.20 |  |
| 3000-5000 | 333 | 97 | 28.36 | 236 | 32.60 |  |
| >5000 | 440 | 137 | 40.06 | 303 | 41.85 |  |
| PrEP motivation | | | | | | |
| Mean (SD) | 40.90 (6.04) | 40.32 (5.95) | | 41.19 (6.07) | | 0.057 |
| Median (IQR) | 42 (37-45) | 41 (36-45) | | 42 (37-46) | |  |

PrEP: pre-exposure prophylaxis; SD: standard deviation; IQR: interquartile range.
